# Supplementary material for: In-House Validation of Multiplex PCR for Simultaneous Detection of Shiga Toxin-Producing Escherichia coli, Listeria monocytogenes and Salmonella spp. in Raw Meats
Source: Foods. 2022 May 25;11(11):1557. doi: 10.3390/foods11111557 (PMC9180326; doi:10.3390/foods11111557)
Supplement: Supplementary file 1 [file foods-11-01557-s001.zip › foods-1718436-supplementary.pdf]

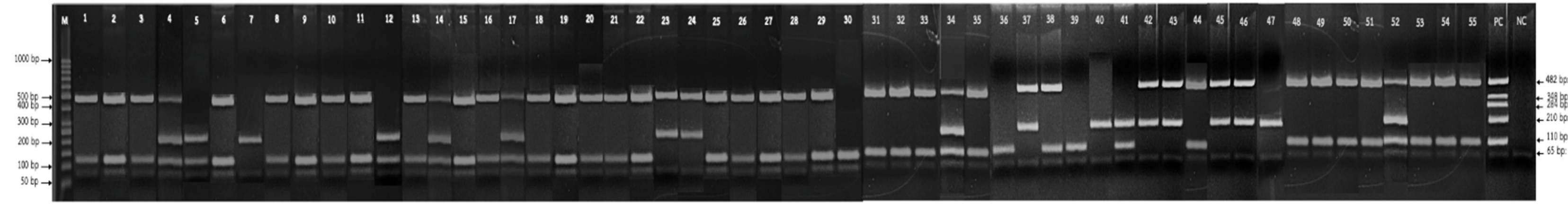

Figure S1 Inclusivity of the developed mPCR assay on agarose gel for specific detection of Shiga toxin-producing *E. coli* strains.
(Lane M, 100 Marker base pair ladder; Lane 1, *E. coli* PSU 4170; Lane 2, *E. coli* PSU 135; Lane 3, *E. coli* PSU 133; Lane 4, *E. coli* serotype O26: H11 CDC 03-3014; Lane 5, *E. coli* PSU 4189; Lane 6, *E. coli* PSU 38; Lane 7, *E. coli* PSU 5023; Lane 8, *E. coli* PSU 4161; Lane 9, *E. coli* PSU 4193; Lane 10, *E. coli* PSU 5027; Lane 11, *E. coli* PSU 4160; Lane 12, *E. coli* PSU 4159; Lane 13, *E. coli* PSU 4167; Lane 14, *E. coli* PSU 4198; Lane 15, *E. coli* PSU 4158; Lane 16, *E. coli* PSU 4171; Land 17, *E. coli* PSU 4195; Lane 18, *E. coli* PSU 4166; Lane 19, *E. coli* PSU 4196; Lane 20, *E. coli* PSU 4154; Lane 21, *E. coli* PSU 4192; Lane 22, *E. coli* PSU 4162; Lane 23, *E. coli* PSU 149; Lane 24, *E. coli* PSU150; Lane 25, *E. coli* PSU 4169; Lane 26, *E. coli* PSU 4155; Lane 27, *E. coli* PSU5029; Lane 28, *E. coli* PSU 4197; Lane 29, *E. coli* PSU 5028; Lane 30, *E. coli* PSU 5030; Lane 31, *E. coli* PSU 4153; Lane 32, *E. coli* PSU 4163; Lane 33, *E. coli* PSU 4172; Lane 34, *E. coli* PSU 3802; Lane 35, *E. coli* PSU 4173; Lane 36, *E. coli* DMST 19341; Lane 37, *E. coli* DMST 30538; Lane 38, *E. coli* DMST 19342; Lane 39, *E. coli* DMST 50661; Lane 40, *E. coli* DMST 48719, Lane 41, *E. coli* DMST 50660, Lane 42, *E. coli* DMST 30539; Lane 43, *E. coli* DMST 19340; Lane 44, *E. coli* DMST 50659, Lane 45, *E. coli* DMST 30536; Lane 46, *E. coli* DMST 30537; Lane 47, *E. coli* DMST 50662; Lane 48, *E. coli* PSU 5026; Lane 49, *E. coli* PSU 4156; Lane 50, *E. coli* PSU 4191; Lane 51, *E. coli* PSU 148; Lane 52, *E. coli* PSU 4190; Lane 53, *E. coli* PSU 4157; Lane 54, *E. coli* PSU 4164; Lane 55, *E. coli* PSU 4165; PC, Positive control (All three target bacteria; *E. coli* CDC 03-3014, *L. monocytogenes* DMST 17303 and *S.* Enteritidis DMST 15676); NC, Negative control (Molecular biology-grade water))

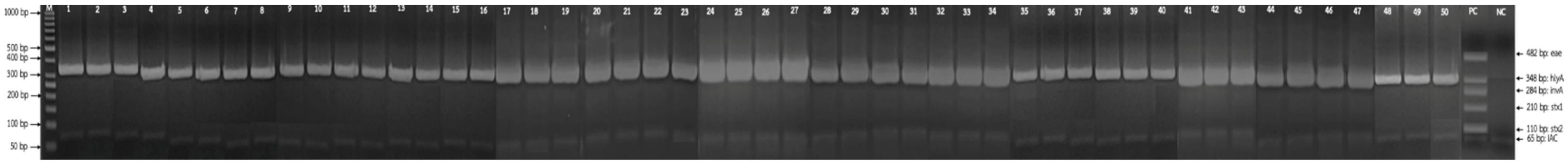

Figure S2 Inclusivity of the developed mPCR assay on agarose gel for specific detection of *L. monocytogenes* strains.

(Lane M, 100 Marker base pair ladder; Lane 1, *L. monocytogenes* ATCC 7644; Lane 2, *L. monocytogenes* Li 23 ATCC 19114; Lane 3, *L. monocytogenes* Li 2107 ATCC 19116; Lane 4, *L. monocytogenes* DMST 41455; Lane 5, *L. monocytogenes* DMST 17303; Lane 6, *L. monocytogenes* DMST 20093; Lane 7, *L. monocytogenes* DMST 20422; Lane 8, *L. monocytogenes* DMST 20423; Lane 9, *L. monocytogenes* DMST 20425; Lane 10, *L. monocytogenes* DMST 21164; Lane 11, *L. monocytogenes* DMST 21165; Lane 12, *L. monocytogenes* DMST 23136; Lane 13, *L. monocytogenes* DMST 23146; Lane 14, *L. monocytogenes* DMST 23150; Lane 15, *L. monocytogenes* DMST 23151; Lane 16, *L. monocytogenes* DMST 23710; Lane 17, *L. monocytogenes* DMST 27738; Lane 18, *L. monocytogenes* DMST 31799; Lane 19, *L. monocytogenes* DMST 31800; Lane 20, *L. monocytogenes* DMST 31801; Lane 21, *L. monocytogenes* DMST 31802; Lane 22, *L. monocytogenes* DMST 31804; Lane 23, *L. monocytogenes* DMST 32862; Lane 24, *L. monocytogenes* DMST 33253; Lane 25, *L. monocytogenes* DMST 36156; Lane 26, *L. monocytogenes* DMST 37884; Lane 27, *L. monocytogenes* DMST 37885; Lane 28, *L. monocytogenes* DMST 41456; Lane 29, *L. monocytogenes* DMST 41457; Lane 30, *L. monocytogenes* DMST 41458; Lane 31, *L. monocytogenes* DMST 41642; Lane 32, *L. monocytogenes* DMST 41647; Lane 33, *L. monocytogenes* DMST 44932; Lane 34, *L. monocytogenes* DMST 44933; Lane 35, *L. monocytogenes* DMST 44934; Lane 36, *L. monocytogenes* DMST 44935; Lane 37, *L. monocytogenes* DMST 44936; Lane 38, *L. monocytogenes* DMST 45433; Lane 39, *L. monocytogenes* DMST 45683; Lane 40, *L. monocytogenes* DMST 45984; Lane 41, *L. monocytogenes* DMST 46456; Lane 42, *L. monocytogenes* DMST 47501; Lane 43, *L. monocytogenes* DMST 47502; Lane 44, *L. monocytogenes* DMST 47503; Lane 45, *L. monocytogenes* DMST 50339; Lane 46, *L. monocytogenes* 100; Lane 47, *L. monocytogenes* 101; Lane 48, *L. monocytogenes* 108; Lane 49, *L. monocytogenes* 310; Lane 50, *L. monocytogenes* Scott A; PC, Positive control (All three target bacteria; *E. coli* CDC 03-3014, *L. monocytogenes* DMST 17303 and *S.* Enteritidis DMST 15676); NC, Negative control (Molecular biology-grade water))

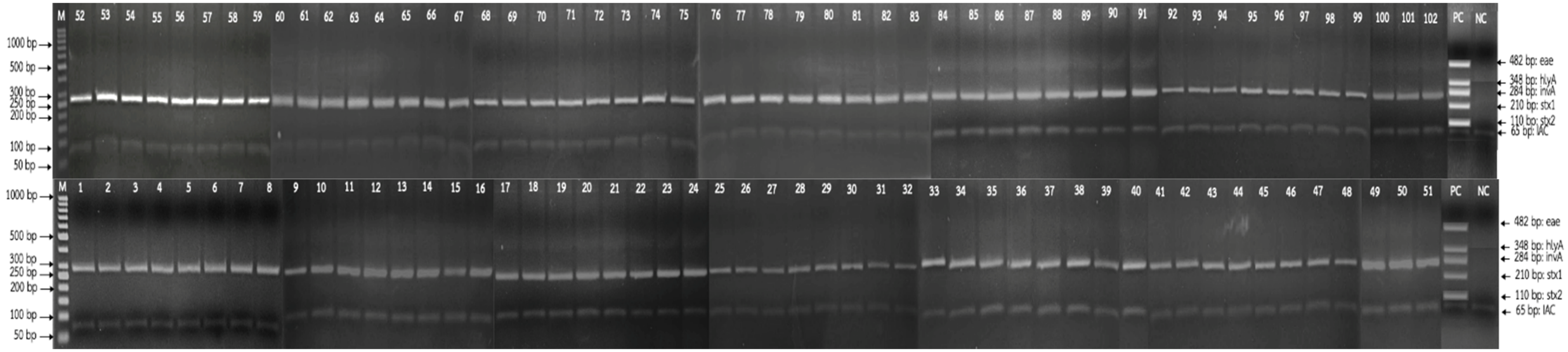

Figure S3 Inclusivity of the developed mPCR assay on agarose gel for specific detection of *Salmonella* strains
(Lane M, 100 Marker base pair ladder; Lane 1, *S.* Aberdeen DMST 19198; Lane 2, *S.* Abony PVKU 1f; Lane 3, *S.* Agona DMST 23970; Lane 4, *S.* Alachua DMST 19203; Lane 5, *S.* Albany DMST 50696; Lane 6, *S.* Altona DMST 62226; Lane 7, *S.* Amsterdam WPKU 1g; Lane 8, *S.* Anatum DMST 50705; Lane 9, *S.* Apeyeme PVKU 2; Lane 10, *S.* Augustenborg DMST 50631; Lane 11, *S.* Bangkok DMST 50834; Lane 12, *S.* Bareilly DMST 62231; Lane 13, *S.* Bergen DMST 19206; Lane 14, *S.* Blockley DMST 16821; Lane 15, *S.* Bongori ATCC 43975; Lane 16, *S.* Bovismorbificans DMST 17379; Lane 17, *S.* Braenderup DMST 62234; Lane 18, *S.* Bredeney PVKU 3; Lane 19, *S.* Canstatt PVKU 4; Lane 20, *S.* Cerro DMST 19200; Lane 21, *S.* Chester WPKU 2; Lane 22, *S.* Chicago KU 1h; Lane 23, *S.* Corvalis KU 2; Lane 24, *S.* Derby DMST 16880; Lane 25, *S.* Dublin WPKU 3; Lane 26, *S.* Eastbourne WPKU 4; Lane 27, *S.* Emek WPKU 5; Lane 28, *S. enterica* subsp. *salamae ser, 17:gt:-* DMST 19207; Lane 29, *S.* Adelaide ATCC 10718; Lane 30, *S.* Bispebjerg ATCC 9842; Lane 31, *S.* Choleraesuis ATCC 6958; Lane 32, *S.* Gaminara ATCC 8324; Lane 33, *S.* Heerlen ATCC 15792; Lane 34, *S.* Hillingdon ATCC 9184; Lane 35, *S.* Illinois ATCC 11646; Lane 36, *S.* Inverness ATCC 10720; Lane 37, *S.* Kirkee ATCC 8322; Lane 38, *S.* Oranienburg ATCC 9239; Lane 39, *S.* Pullorum ATCC 9120; Lane 40, *S.* Simsbury ATCC 12004; Lane 41, *S.* Vellore ATCC 15611; Lane 42, *S.* Zwickau ATCC 15805; Lane 43, *S.* Dar-es-salaam ATCC 6959; Lane 44, *S.* Hooggraven ATCC 15786; Lane 45, *S.* Enteritidis DMST 15676; Lane 46, *S.* Falkensee DMST 50716; Lane 47, *S.* Fresno DMST 19197; Lane 48, *S.* Give DMST 50827 G; Lane 49, *S.* Hadar DMST 32769; Lane 50, *S.* Havana DMST 50710; Lane 51, *S.* Heidelberg PVKU 5; Lane 52, *S.* Hvittingfoss DMST 62220; Lane 53, *S.* Indiana PVKU 6; Lane 54, *S.* Infatis PVKU 7; Lane 55, *S.* I4,12:i:- PVKU 8; Lane 56, *S.* Johnnesburg DMST 50835; Lane 57, *S.* Kedougou DMST 33890; Lane 58, *S.* Kentucky DMST 50701; Lane 59, *S.* KiamBu PVKU 9; Lane 60, *S.* Krefeld DMST 62227; Lane 61, *S.* Lexington DMST 50707; Lane 62, *S.* Liverpool PVKU 10; Lane 63, *S.* Livingstone DMST 50633; Lane 64, *S.* London DMST 62232; Lane 65, *S.* Manhattan PVKU 11; Lane 66, *S.* Matopeni DMST 62218; Lane 67, *S.* Mbandaka DMST 62238; Lane 68, *S.* Minnesota KU 3; Lane 69, *S.* Molade PVKU 12; Lane 70, *S.* Montevideo PVKU 13; Lane 71, *S.* Moscow PVKU 14; Lane 72, *S.* Muenchen PVKU 15; Lane 73, *S.* Muenster DMST 62235; Lane 74, *S.* Newport DMST 15675; Lane 75, *S.* Orion WPKU 6; Lane 76, *S.* Oslo WPKU 7; Lane 77, *S.* Ouakam DMST 50824; Lane 78, *S.* Panama DMST 50703; Lane 79, *S.* Paratyphi A DMST 15673; Lane 80, *S.* Paratyphi B WPKU 8; Lane 81, *S.* Paratyphi C WPKU 9; Lane 82, *S.* Poona KU 4; Lane 83, *S.* Ramat-gan WPKU 10; Lane 84, *S.* Rissen DMST 16876; Lane 85, *S.* Rubislaw DMST 62223; Lane 86, *S.* Saintpaul DMST 62225; Lane 87, *S.* Schwarzengrund WPKU 11; Lane 88, *S.* Typhi WPKU 12; Lane 89, *S.* Singapore DMST 50636; Lane 90, *S.* Stanley DMST 33894; Lane 91, *S.* Tennessee WPKU 13; Lane 92, *S.* Thempson PVKU 16; Lane 93, *S.* Typhimurium DMST 562; Lane 94, *S.* Brunei KU 5; Lane 95, *S.* Virchow DMST 16857; Lane 96, *S.* Wandsworth DMST 19204; Lane 97, *S.* Warthington DMST 33889; Lane 98, *S.* Waycross DMST 19205; Lane 99, *S.* Weltevreden DMST 16820; Lane 100, *S.* Urbana PVKU 17; Lane 101, *S.* Soerenga PVKU 18; Lane 102, *S.* Weston PVKU 19; PC, Positive control (All three target bacteria; *E. coli* CDC 03-3014, *L. monocytogenes* DMST 17303 and *S.* Enteritidis DMST 15676); NC, Negative control (Molecular biology-grade water))

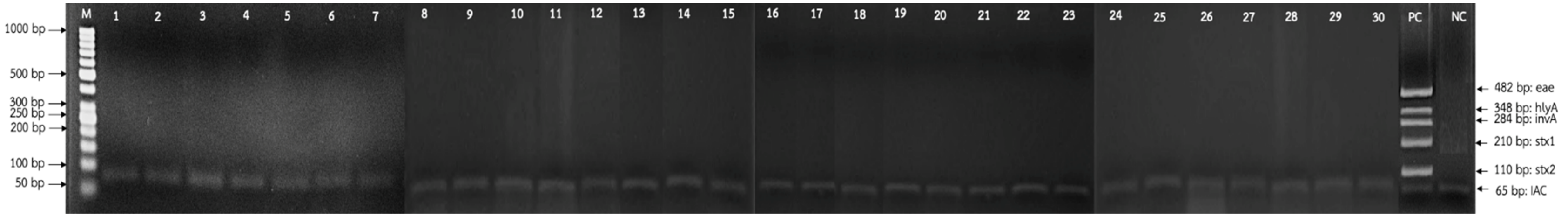

Figure S4 Exclusivity of the developed mPCR assay on agarose gel for specific detection of non-target bacteria.
(Lane M, 100 Marker base pair ladder; Lane 1, *Aeromonas hydrophila* DMSTa 21250; Lane 2, *Bacillus cereus* DMST 5040; Lane 3, *Campylobacter coli* DMST 18034; Lane 4, *Campylobacter jejuni* DMST 15190; Lane 5, *Citrobacter freundii* DMST 16368; Lane 6, *Cronobacter sakazakii* DMST 17894; Lane 7, *Enterobacter cloacae* DMST 434; Lane 8, *Enterococcus faecalis* DMST 4736; Lane 9, *Klebsiella pneumonia* DMST 8216; Lane 10, *Lactococcus lactis* KU 6b; Lane 11, *Lactobacillus brevis* KU 7; Lane 12, *Listeria innocua* DMST 9011; Lane 13, *Listeria ivanovii* DMST 9012; Lane 14, *Pediococcus pentosaceus* DMST 18752; Lane 15, *Proteus vulgaris* DMST 557 C; Lane 16, *Pseudomonas aeruginosa* DMST 4739; Lane 17, *Pseudomonas fluorescens* KU 8; Lane 18, *Shigella dysenteriae* DMST 15111; Lane 19, *Shigella flexneri* DMST 4423; Lane 20, *Shigella sonnei* DMST 561; Lane 21, *Staphylococcus aureus* DMST 8840; Lane 22, *Streptococcus pyogenes* DMST 30653; Lane 23, *Streptococcus suis* serotype II DMST 18783; Lane 24, *Vibrio cholerae* nonO1 /nonO139 DMST 2873; Lane 25, *Vibrio parahaemolyticus* DMST 21243; Lane 26, *Vibrio vulnificus* DMST 21245; Lane 27, *Yersinia enterocolitica* DMST 8012; Lane 28, *Yersinia pseudotubercolosis* DMST 16385; Lane 29, *Bacillus subtilis* (Ehrenberg) Cohn BCC 6327; Lane 30, *Staphylococcus epidermidis* KU 9; PC, Positive control (All three target bacteria; *E. coli* CDC 03-3014, *L. monocytogenes* DMST 17303 and *S.* Enteritidis DMST 15676); NC, Negative control (Molecular biology-grade water)).
